# Out of Touch? Visual Load Induces Inattentional Numbness

**DOI:** 10.1037/xhp0000218

**Published:** 2016-03-14

**Authors:** Sandra Murphy, Polly Dalton

**Affiliations:** 1 Department of Psychology, Royal Holloway, University of London

**Keywords:** inattentional numbness, tactile awareness, perceptual load, multisensory processing

## Abstract

It is now well known that the absence of attention can leave people unaware of both visual and auditory stimuli (e.g., Dalton & Fraenkel, 2012; Mack & Rock, 1998). However, the possibility of similar effects within the tactile domain has received much less research. Here, we introduce a new tactile inattention paradigm and use it to test whether tactile awareness depends on the level of perceptual load in a concurrent visual task. Participants performed a visual search task of either low or high perceptual load, as well as responding to the presence or absence of a brief vibration delivered simultaneously to either the left or the right hand (50% of trials). Detection sensitivity to the clearly noticeable tactile stimulus was reduced under high (vs. low) visual perceptual load. These findings provide the first robust demonstration of “inattentional numbness,” as well as demonstrating that this phenomenon can be induced by concurrent visual perceptual load.

In a world rich with sensory information, we are unable to perceive everything around us. Selective attention thus plays a crucial role in cognition, prioritizing information most relevant to our current needs. This interplay between perception and attention is strikingly demonstrated in the inattention literature, which demonstrates that salient yet unexpected changes can go unnoticed when attention is focused elsewhere. These phenomena have been demonstrated in vision (e.g., Drew, Võ, & Wolfe, 2013; Simons & Chabris, 1999) and hearing (Dalton & Fraenkel, 2012; Dehais et al., 2014; Koreimann, Gula, & Vitouch, 2014). However, the role of attention in tactile awareness has received little focus. This is nevertheless an important issue, because the tactile modality is sometimes considered more “primitive” than vision and hearing (because incoming tactile information is directly informative, whereas visual or auditory stimuli need further processing before identification occurs; Gregory, 1967), suggesting that tactile perception might be less open to inattentional failures than other senses.

Only one preliminary investigation into “inattentional numbness” has been reported (Mack & Rock, 1998; although see Gallace, Tan, & Spence, 2006, and Pritchett, Gallace, & Spence, 2011, for reports of tactile change blindness). Participants identified letters drawn on one arm, and, on the critical trial, a water droplet or air puff was concurrently delivered to the unattended arm. When asked immediately afterward whether they had noticed anything other than the target, 60% of participants were unaware of the water droplet and 90% were unaware of the air puff. However, because these results relied on retrospective questioning, they may reflect memory failures rather than absence of perception. For this reason, recent experiments in this area typically measure detection sensitivity to a frequently appearing critical stimulus, rather than relying on retrospective reports of one-off unexpected events (e.g., Macdonald & Lavie, 2008; Raveh & Lavie, 2015). In addition, the letter traces, water droplets, and air puffs in Mack and Rock’s (1998) experiments were delivered manually and thus uncontrolled. This makes the results unreliable, as acknowledged by the authors (“given the rather crude equipment and procedure, these results are at best only suggestive”; p. 224). For example, the experimenters might have unwittingly affected the results through subtle differences in their behavior (leading to differences in stimulus presentation) on different types of trials. Thus, although these preliminary findings are encouraging in suggesting that tactile stimuli may remain undetected in the absence of attention, they did not provide a robust demonstration. Here, we introduce a new detection sensitivity measure of inattentional numbness using highly controlled tactile stimuli, allowing us to provide the first robust investigation of this phenomenon.

Within the visual domain, people’s ability to notice unexpected, task-irrelevant stimuli has been shown to decrease as the perceptual load of the task at hand increases (e.g., Cartwright-Finch & Lavie, 2007; Macdonald & Lavie, 2008). A focal task of low perceptual load is argued to leave “spare” capacity for processing additional stimuli, whereas a focal task of higher perceptual load does not (e.g., Lavie, 1995). These arguments are now well established within the visual domain; however, some intriguing recent research has also suggested that visual perceptual load determines auditory detection sensitivity. For example, Macdonald and Lavie (2011) demonstrated reduced awareness of a one-off, unexpected auditory event under high (vs. low) visual perceptual load. Furthermore, Raveh and Lavie (2015) reported a modulation by visual perceptual load of detection sensitivity to a repeatedly presented auditory stimulus. These demonstrations extend the principles of load theory to audiovisual settings, suggesting that processing capacity is shared between these modalities. However, the question of whether similar effects can operate between vision and touch remains open. These issues also have relevance for applied settings. For example, given the recent surge in tactile alerting of drivers (see Meng & Spence, 2015, for a review), the question of whether tactile events can go unnoticed when attention is focused on a perceptually demanding visual scene is particularly pertinent. Thus, a second focus of the present experiment is to ask whether increasing the perceptual demands of an attended visual task can reduce sensitivity for a concurrent tactile event. Rather than measuring awareness of a one-off unexpected event (e.g., Mack & Rock, 1998), we measured detection sensitivity for a tactile stimulus presented with high frequency (50% of trials). Participants performed a visual search task of either high or low perceptual load, and immediately after response reported the presence or absence of the tactile stimulus.

## Method

### Participants

Sixteen participants recruited at Royal Holloway University of London, Egham, took part in exchange for £8 ($11). Two were replaced because of detection accuracy <75% in the control condition. For those included, the average age was 21 years (ranging from 18 to 36). Three participants were male, and one (female) was left-handed. All reported normal or corrected-to-normal vision and no haptic impairment.

### Apparatus and Stimuli

The experiment was programmed and run using E-prime 2.0 (Psychology Software Tools Inc., 2012), presented on a 19-in. Samsung SyncMaster 940N monitor (60-Hz refresh rate). Responses to the visual task and the tactile event were made using two foot pedals (Psychology Software Tools Inc). Participants sat at a viewing distance of 57 cm from the screen. Tactile stimuli consisted of sound files delivered through two Starkey bone conduction hearing aids attached with surgical tape to the left and right palms. Participants sat with their hands stretched out in from of them on a foam board (with hollowed-out slots to ensure 10 cm separation) with the palms faced upward. The hands were covered with a black cloth to conceal any visual information of the tactile event. White noise was presented via ProSound headphones at approximately 56 dB SPL to mask the sound from the tactile stimulators.

The visual task consisted of six letters forming a circle with a radius of 1.9° visual angle, presented at fixation in gray on a black background. The target letter “X” or “N” (0.6° × 0.6°) appeared with equal likelihood in one of the six locations (each equally likely). Under low perceptual load, the remaining locations consisted of “o”s (0.4° × 0.4°), whereas under high load, five out of a pool of six letters (H, K, M, V, W, and Z; same size as target letters) were randomly allocated to the remaining spaces.

The critical stimulus consisted of a 20-ms vibration (square wave tone, 100 Hz) occurring 50 ms after the onset of the letter display. The critical stimulus was presented on 50% of trials and appeared with equal likelihood on either the left or the right hand.

### Procedure

**Figure 1 fig1:**
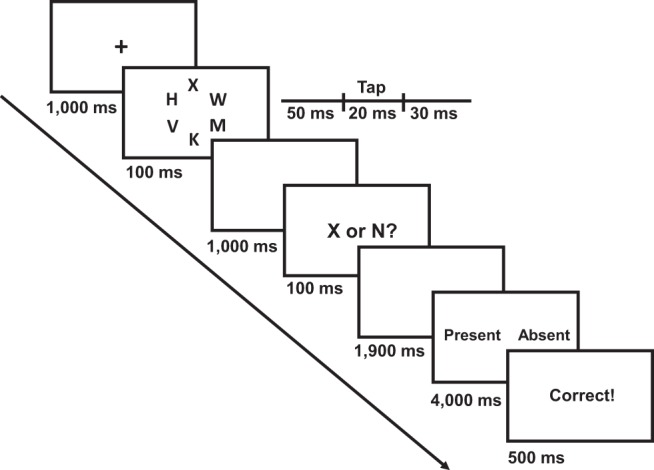
Illustration of a high perceptual load trial in which the tactile stimulus was present (50% of trials, left or right hand with equal likelihood). Under low perceptual load, the target (X or N) was presented among “o”s.

Figure 1 presents a timeline of an example trial. Trials began with a central fixation cross, presented for 1,000 ms, followed by the letter circle (and critical stimulus, if present) for 100 ms. The task response was delayed with a 1,000-ms blank screen. Following Macdonald and Lavie (2008, Experiment 2), this was to rule out any differences in preparation time for the tactile response because of slower reaction times (RTs) under high versus low load. Subsequently “X or N?” was displayed on the screen for 100 ms, followed by a 1,900-ms blank screen (total response window of 2,000 ms). Half of the participants lifted the left foot pedal for X and the right for N, whereas the other half had the reverse response pattern. Once the response window had passed (regardless of when or if a response was made), the words “Present” and “Absent” were presented for up to 4,000 ms, one to the left and one to the right. Participants lifted the corresponding pedal, at which point feedback for the target performance was presented for 500 ms. The present–absent and left–right pedal correspondence changed every block to eliminate any systematic differences in responses as a function of the target identity (which had a constant response mapping).[Fig-anchor fig1]

Participants were shown 12 slowed-down example trials, verbally confirming whether the critical stimulus was presented (50% present). If they failed to detect this more than three times, the examples were repeated. Two practice blocks followed, of 12 trials each, one of high and one of low perceptual load. Participants then performed four blocks of 48 trials each, alternating between low and high load in an ABBA/BAAB fashion. Finally, two control blocks of 48 trials each were presented, one of high and one of low load. Here, participants were instructed to ignore the letters and only focus on whether the critical stimulus was present or absent.

## Results

### Visual Task

Only correct trials with RTs <1,500 ms were included in the RT analysis. As predicted, because participants had to delay their responses, there was no difference in RTs between low (*M* = 426 ms) and high (*M* = 439 ms) perceptual load, *F* < 1. This is important in eliminating the possibility that any modulation of tactile awareness by visual perceptual load could relate to the tactile detection responses being delayed by different amounts under different load conditions. Nevertheless, mean accuracy was lower under high (*M* = 75%) versus low load (*M* = 96%), *F*(1, 15) = 141, *p* < .0001, η_p_^2^ = .904, indicating that our load manipulation was successful.

### Tactile Detection

Only trials with a correct visual task response were included in the tactile detection analysis. Mean percent detection accuracy, sensitivity (*d*′), and response bias (β) were calculated as a function of perceptual load, presented in Table 1 (hit and false alarm rates are also included for reference). Detection accuracy was lower under high (vs. low) load, *F*(1, 15) = 30.92, *p* < .0001, η_p_^2^ = .673. Similarly, *d*′ was lower under high (vs. low) load, *F*(1, 15) = 34.81, *p* < .0001, η_p_^2^ = .699, indicating that increasing visual perceptual load reduced awareness of the critical tactile stimulus. Response bias (β) did not change as a function of load (*F* < 1), implying that participants did not become more lenient with a more perceptually demanding visual task. This was true despite the increase in false alarms under high versus low perceptual load (see Table 1).[Table-anchor tbl1]

Similar to previous reports (Macdonald & Lavie, 2008; Raveh & Lavie, 2015), we repeated this analysis including incorrect visual task responses to address the possibility that the reduced detection sensitivity under high (vs. low) load may have been because of the reduced number of trials included in the analysis for that condition (because of the higher error rates). Detection accuracy was lower under high load (*M* = 78%) compared with low load (*M* = 88%), *F*(1, 15) = 27.16, *p* < .0001, η_p_^2^ = .644. Similarly, *d*′ was lower under high load (*M* = 1.93) than under low load (*M* = 2.70), *F*(1, 15) = 31.02, *p* < .0001, η_p_^2^ = .674. Again, β was no different between high (*M* = −.11) and low (*M* = −.09, F < 1) load, suggesting that response bias did not change as load increased. These findings support the original analysis, ruling out any influence of reduced trial numbers under high (vs. low) load.

Detection accuracy in the control blocks (in which participants only responded to the tactile stimulus, while ignoring the visual task) was no different between high (*M* = 97%) and low (*M* = 98%) perceptual load, *F*(1, 15) < 1. Similarly, *d*′ and β were no different between high (*d*′, *M* = 3.71; β, *M* = .25) and low (*d*′, *M* = 3.63; β, *M* = −.11) load, *F*(1, 15) < 1 (*d*′), *F*(1, 15) = 3.22, *p* = .093, η_p_^2^ = .177 (β). The lack of any perceptual load effects in the control blocks suggests that the reduced detection sensitivity as a function of load in the experimental blocks relates to the modulation of task demands rather than the visual display properties. The near-perfect performance in these control blocks also demonstrates that the tactile stimulus was clearly detectable under conditions of full attention.

## Discussion

These findings provide the first robust demonstration of inattentional numbness, whereby awareness of a tactile event is reduced when attention is focused elsewhere. Thus, despite the more directly informative nature of tactile stimulation (compared, e.g., with visual or auditory stimuli, whose identification requires substantial further processing; Gregory, 1967), tactile perception is still susceptible to inattentional failures to an extent that is comparable with the other senses.

Critically, the magnitude of inattentional numbness observed in this experiment was modulated by the perceptual demands of a concurrent visual task, such that an increase in visual perceptual load reduced tactile detection sensitivity. Importantly, this extends perceptual load theory to the visuotactile domain, suggesting that processing capacity is to some extent shared between vision and touch.

We acknowledge that our claims currently hinge on the single experiment presented here. Nevertheless, given the clear strength of the effects observed, and the fact that the visual task used is a widely replicated manipulation of perceptual load—which has also been demonstrated to reduce both visual (Macdonald & Lavie, 2008) and auditory detection sensitivity (Raveh & Lavie, 2015)—we argue that the results are sufficiently robust and reliable to warrant such conclusions.

Our claims are also in line with the findings of previous research. For example, Santangelo and Spence (2007) observed attentional capture by an irrelevant tactile stimulus under conditions of low concurrent visual demand (in the absence of an rapid serial visual presentation (RSVP) stream), which was eliminated as the visual demands increased (through the introduction of an RSVP stream). Similarly, Jones and Forster (2013) demonstrated that event-related potential components such as the late somatosensory negativity—reflecting anticipatory activity in the somatosensory regions prior to a tactile event—were reduced when performing a concurrent visual task (vs. just performing the tactile task). The current findings converge with this evidence to suggest that ongoing visual demands can modulate tactile processing. However, whereas the previous research demonstrated this as a function of the presence (vs. absence) of any visual demand, the current experiment demonstrates similar findings through modulation of demand in a visual task that is performed in both conditions. This is important in ruling out potential effects of strategy and perceptual organization that can arise in designs comparing single and dual task performance. The current findings also converge with previous demonstrations of reduced pain perception under high (vs. low) visual perceptual load (Romero, Straube, Nitsch, Miltner, & Weiss, 2013; Veldhuijzen, Kenemans, de Bruin, Olivier, & Volkerts, 2006). However, these studies relied on subjective pain ratings, which may be open to alternative explanations such as task demands. It would be an interesting future direction to investigate more directly whether visual perceptual load can reduce awareness of painful stimuli by measuring detection sensitivity.

The fact that visual perceptual load can determine tactile processing chimes with findings demonstrating modulations by visual perceptual load of auditory processing (e.g., Raveh & Lavie, 2015). Interestingly however, auditory perceptual load does not seem to influence visual processing (Rees, Frith, & Lavie, 2001), nor auditory processing (Murphy, Fraenkel, & Dalton, 2013), which Murphy et al. (2013) suggested may be related to the nature of the different selection mechanisms operating in vision and in hearing. An important next question therefore concerns whether tactile perceptual load can modulate tactile awareness, as well as awareness in other senses. If this were not found to be the case, this would suggest that the principles of perceptual load might be limited to contexts involving a visual focal task.

The current findings of reduced tactile sensitivity with increased visual demands could have important applications—for instance, in relation to the growing use of tactile warning information in both cars (see Meng & Spence, 2015) and aircraft (e.g., van Veen & van Erp, 2001). In particular, the increase in false alarm rates, but not response criterion, under high perceptual load seems to reflect a reduced ability to determine whether or not the tactile stimulus had been present. This could be as detrimental to performance as missing an alerting signal (e.g., Dixon, Wickens, & McCarley, 2007). However, tactile warning systems are likely to deliver information less frequently (Meng & Spence, 2015) and with less emphasis than in the present experiment. It is thus important to investigate whether false alarm rates are reduced if the tactile stimuli occur infrequently and if participants only report their presence (rather than being probed on every trial).

In conclusion, this is the first experiment to provide a robust demonstration of inattentional numbness. It also demonstrates a reduction in tactile detection sensitivity with increasing visual perceptual load in the relevant task. Importantly, this suggests that perceptual resources may be shared between the visual and tactile modalities.

## Figures and Tables

**Table 1 tbl1:** Hit Rate, False Alarms, Detection Accuracy, D′, and β as a Function of Perceptual Load (Low, High)

Perceptual load	Hits (%)	False alarms (%)	Detection accuracy (%)	*d*′	β
Low	91	14	89	2.84	−.17
High	88	31	79	1.99	−.15
